# Sex differences in gout characteristics: tailoring care for women and men

**DOI:** 10.1186/s12891-017-1465-9

**Published:** 2017-03-14

**Authors:** Leslie R. Harrold, Carol J. Etzel, Allan Gibofsky, Joel M. Kremer, Michael H. Pillinger, Kenneth G. Saag, Naomi Schlesinger, Robert Terkeltaub, Vanessa Cox, Jeffrey D. Greenberg

**Affiliations:** 1 Department of Medicine and Orthopedics, University of Massachusetts Medical School, 55 Lake Avenue North, Worcester, MA 01655 USA; 2 Corrona, LLC, Southborough, MA USA; 3 Department of Epidemiology, UT MD Anderson Cancer Center, Houston, TX USA; 4 Hospital for Special Surgery-Weill Medical College of Cornell University, New York, NY USA; 5 Albany Medical College and The Center for Rheumatology, Albany, NY USA; 6 NYU School of Medicine, New York, NY USA; 7 University of Alabama at Birmingham, Birmingham, AL USA; 8 Rutgers-Robert Wood Johnson Medical School, New Brunswick, NJ USA; 9 VA Medical Center, UCSD, San Diego, CA USA

**Keywords:** Gout, Comorbidity, Gender, Quality of Care

## Abstract

**Background:**

To characterize the differences between women and men with gout.

**Methods:**

We analyzed a US national cohort of gout patients cared for by rheumatologists.

**Results:**

Compared with the 1012 men with gout, women with gout (*n* = 262) were older (71 vs. 61 years, *p* < 0.001) and had a greater burden of comorbid conditions (*p* < 0.001 for hypertension, diabetes, renal disease and obesity). Risk factors for gout differed with women more often taking diuretics (*p* < 0.001), while men more frequently had dietary triggers (*p* < 0.05).

**Conclusions:**

The profiles of women and men with gout are markedly different, suggesting a need to tailor treatment recommendations.

**Electronic supplementary material:**

The online version of this article (doi:10.1186/s12891-017-1465-9) contains supplementary material, which is available to authorized users.

## Background

Gout is the most common inflammatory arthritis, affecting approximately 8.3 million Americans (6.1 million men and 2.2 million women) [1]. Gout prevalence has been increasing over the last half century, due to the aging of the population, increased use of medications that can trigger gout, and the obesity epidemic [2]. Gout is associated with a reduced quality of life, functional impairment, reduced productivity and a higher risk of death [3, 4]. Accumulating evidence suggests that gout is an independent risk factor for cardiovascular morbidity and mortality in both women and men [5].

While gout has been studied for centuries, most research has focused on men. For example, there have been very few studies that have examined the epidemiology of gout based on sex. Those that have included relatively small numbers of women [6, 7] or lacked gout-specific details [8]. Even clinical trials of therapeutic agents for the acute and chronic management of gout included very few women [9–11]. Given the prevalence and impact of gout, it is essential to identify the factors that impede optimal gout management in both women and men in order to provide tailored treatment recommendations.

In 2012, CORRONA (the Consortium of Rheumatology Researchers of North America) created a national gout registry in which rheumatologists enrolled their gout patients with longitudinal assessment. Within this setting, our objective was to compare women and men with gout to see whether they differed in terms of comorbid conditions associated with gout and risk factors for gout flares. We hypothesized that the patient profile of women with gout would be very different as compared to men with the condition.

## Methods

### Data source and population

CORRONA creates prospective observational cohorts of arthritis patients enrolled by participating rheumatologists in both academic and private practice sites. There were 1273 patients with rheumatologist-diagnosed gout based on the 1977 diagnostic criteria [12] (the registry predated the 2015 American College of Rheumatology Gout Classification criteria) and enrolled in the CORRONA registry database between 11/1/2012 and 3/31/2014 from 34 rheumatology practices across 20 states with 76 participating rheumatologists. Approvals for data collection and analyses were obtained for academic and private practice sites from local and central institutional review boards, respectively.

### Measures and data collection

Data were collected from patients and their treating rheumatologists using standard clinical research forms based on the American College of Rheumatology [13] treatment recommendations. Information collected included demographics, comorbid conditions, gout presentation, disease severity and activity, family history, body mass index (BMI), dietary intake over the past week, use of medications that can raise serum urate level (e.g., diuretics), use of medications for acute gouty inflammation (nonsteroid anti-inflammatory drugs [NSAIDs], colchicine, steroids) and urate-lowering therapy (ULT) including uricosurics, xanthine oxidase inhibitors, and recombinant uricase [pegloticase]. Documentation included physician exam findings of tophi and inflamed joints, physician and patient global assessments of disease activity, patient assessment of pain, the Health Assessment Questionnaire (HAQ) assessing physical function, and serum urate levels (SUA) from laboratory tests obtained within 10 days of the clinical encounter (this data is not mandated by the study protocol). Patients reported the number of days in the past 3 months there were unable to perform their usual activity. Health care utilization data, including gout hospitalizations within the last 3 years as well as Emergency Room and/or outpatients visits in the past 12 months for gout flares, were gathered. Additionally patients reported how many flares they managed themselves without seeing a health care professional.

### Comparison by sex

We compared patients’ baseline characteristics at the time of enrollment, including demographics, gout-related comorbid conditions and medications, gout characteristics, contributing medical conditions and medications. We compared dietary factors associated with gout flares in men and women in terms of any intake over the past week of beef, pork, seafood and alcohol, as well as the number of weekly servings consumed. We also evaluated rate of use of ULT in the subset of subjects who met ACR criteria for urate lowering (e.g., 2 or more gout flares/year, presence of tophi, history of kidney stones or presence of chronic kidney disease (CKD; stage 3 or greater).

### Statistical analysis

We use a cross-sectional study design, evaluating patients at the time of enrollment. Descriptive statistics were performed using t tests and chi square tests or Fisher exact tests as appropriate. As a sensitivity analysis, when examining the burden of comorbidity between the 2 sexes, we used 3 propensity score (PS) approaches to balance the differences in the ages and disease duration of the women and men with gout. We conducted these sensitivity analyses to address a potential concern that differences in comorbidity burden could have been related to sex differences in age (women were on average 10 years older than men) or disease duration (men had on average 5 more years of gout) The PS approaches allow us to compare women and men with similar demographic profiles. We used 3 approaches to assess the robustness of our findings. For approach #1, we derived a PS using age and disease duration and used PS trimming to excluded patients with a PS score in the <5 percentile and >95 percentile range. For approach #2, we included only those patients who fell inside the area of common support using age and disease duration separately and then used PS trimming. Approach #3 included only those patients with a conservative common support with PS trimming so that the resulting age and disease duration characteristics of the women and men with gout were within 3 years. All 3 approaches gave similar results to the unadjusted analyses. Given the reduction in the sample size with each of these 3 approaches, we show the unadjusted results in the body of the manuscript but provide the results of the PS approaches in Additional file 1: Table S1. To explore if there were dietary differences between men and women, we conducted adjusted logistic (yes/no dietary intake) and ordinal logistic regression models (0, 1, 2+ servings per week of dietary intake) controlling for age, BMI, duration of gout, comorbidity burden [hypertension, diabetes, renal disease, hyperlipidemia], HCTZ use, other diuretic use and current use of a urate-lowering drug. Sex based comparison for functional status was adjusted for age and presented herein. Lastly ULT use was explored in those who met criteria for treatment based on 2 or more attacks in 12 months or presence of tophi (dosages provided in Additional file 2: Table S2).

## Results

**Fig. 1:**
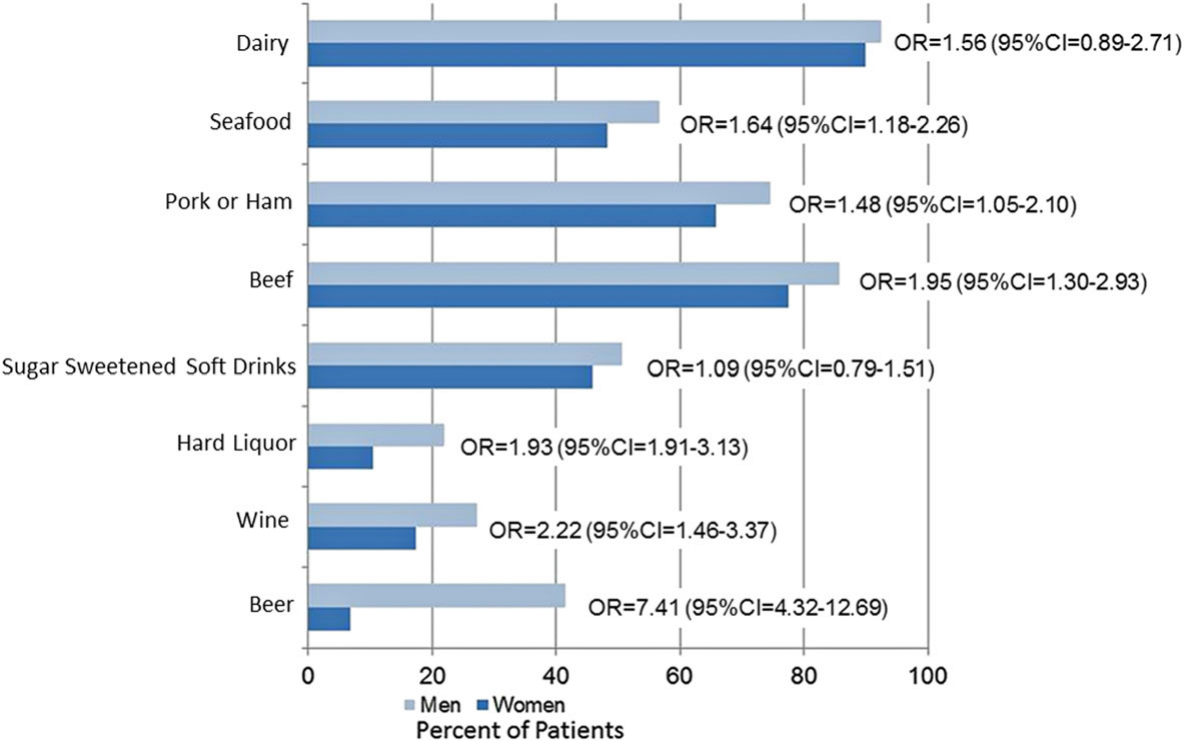
Comparison of the dietary factors that influence gout in women and men. The results are adjusted for age, BMI, duration of gout, comorbidity burden [hypertension, diabetes, renal disease, hyperlipidemia], HCTZ use, other diuretic use and current use of a urate-lowering drug

There were 262 women and 1011 men enrolled into the registry meeting ACR criteria for gout [12] during the study time period. Women were approximately 10 years older than the men (71.2 vs. 60.9, *p* < 0.0001; Table 1). Additionally, women were less likely to be white (88% vs. 94%, *p* = 0.002) or to have private insurance (58% vs. 75%, *p* < 0.001). The overall burden of rheumatologist reported comorbid conditions was greater in women in both the overall cohort (Table 1) and PS trimmed subsets (Additional file 1: Table S1). Women were more impaired in terms of days being unable to perform usual work and activities as well as the HAQ score using age adjusted estimates. Activity and HAQ discrepancies persisted after adjusting for comorbidity and duration of gout (Additional file 1: Table S1). Obesity and diuretic use were also more common in women. Gout characteristics at initial clinical presentation (data not shown) were similar with respect to podagra, monoarthritis, and polyarthritis. Additionally, both the maximum and most recent serum sUA levels were similar, as well as the prevalence of tophi, flare frequency and gout related health care utilization. Women’s gout was less likely to be crystal proven (25% vs. 34%, *p* = 0.004). Patterns of dietary intake differed based on sex. Men were more likely to report intake of beef, pork, seafood, beer, wine and hard liquor in unadjusted and adjusted analyses (Fig. 1) as well as a greater number of servings of those items (Additional file 3: Figure S1).

**Table 1:** Baseline demographic and gout characteristics

Characteristics	Women	Men	P value
	*N* = 262	*N* = 1011	
Demographics			
Age (mean years, SD)	71.2 (±10.7)	60.9 (±13.5)	<0.0001
Race (White n, %)	219 (84)	897 (89)	0.024
Private insurance (n, %)	152 (58)	757 (75)	<0.001
Comorbid conditions			
BMI (mean, SD)	33.5 (±7.8)	31.9 (±6.5)	0.001
Osteoarthritis (n, %)	120 (46)	256 (25)	<0.001
Other inflammatory arthritis (n,%)	19 (7)	34 (3)	<0.001
Medical comorbidities (n,%)			
Heart disease	41 (16)	147 (14)	0.652
Hypertension	201 (77)	574 (57)	<0.001
Diabetes mellitus	73 (28)	168 (17)	<0.001
Renal disease	64 (24)	136 (13)	<0.001
Hyperlipidemia	120 (46)	394 (39)	0.045
Peripheral arterial disease	2 (1)	13 (1)	0.485
Functional Status (mean, SD)			
Number of days unable to do usual work/activities in the prior 3 months	11.1 (±23.7)	6.9 (±195)	0.003
Health Assessment Questionnaire			
Age adjusted means	0.59 (±0.11)	0.49 (±0.13)	<0.001
Medications that may trigger gout (n, %)			
Aspirin	76 (29)	285 (28)	0.794
HCTZ	51 (20)	105 (10)	<0.001
Diuretics other than HCTZ	83 (32)	116 (11)	<0.001
Gout characteristics			
Family history (n, %)	180 (69)	682 (67)	0.701
Crystal proven (n, %)	65 (25)	346 (34)	0.004
Duration of gout (mean, SD)	6.1 (±7.1)	11.0 (±9.8)	<0.0001
Maximum serum uric acid (mean, SD)	8.8 (±2.3)	8.7 (±2.1)	0.481
Current serum uric acid (mean, SD)^a^	5.9 (±2.6)	6.0 (±1.9)	0.449
Presence of tophi (n, %)	60 (23)	224 (22)	0.791
Contraindications for treatment with:	11 (10)	66 (16)	0.0.170
NSAIDS	80 (31)	206 (20)	<0.001
Colchicine	32 (12)	61 (6)	0.001
Flare frequency in prior 12 months (mean, SD)	3.5 (±6.4)	3.7 (±8.2)	0.692
Health care utilization due to gout (n, %)			
Proportion of patients with hospitalizations for gout in the past 3 years	7 (3)	19 (2)	
Proportion with an ER visit for a gout flare in the past 12 months	37 (14)	118 (12)	0.275
Proportion with an outpatient physician visit for a gout flare in the past 12 months	124 (48)	479 (48)	0.918
Frequency of health care utilization for a flare in the prior 12 months:			
ER visits (mean, SD)	0.32 (±1.17)	0.26 (1.49)	0.560
Outpatient encounters (mean, SD)	1.40 (±2.64)	1.30 (±2.68)	0.613

^a^laboratory data were available for 215 women and 870 men with gout

Medications including colchicine, NSAIDs, and glucocorticoids, which can be used either for an acute gouty flare or for prophylaxis when initiating a ULT, were prescribed at similar rates in women and men (Table 2). In addition, over-the-counter supplements for gout were used in both women and men. However, women had greater use of acetaminophen and opioid analgesics. Among those who met criteria for ULT, women were as likely as men to receive it in both unadjusted (77% vs. 83%, *p* = 0.10) or PS adjusted (OR 0.70, 95% confidence interval 0.43–1.15) analyses. Women were more likely to receive febuxostat, while men more likely allopurinol (Table 2).

**Table 2:** Medications and supplements to treat gout

Medication^a^	Women	Men	P value
	*N* = 262	*N* = 1012	
Acute/prophylactic gout medications (n, %)			
Colchicine	86 (33)	366 (36)	0.314
Oral steroids	34 (13)	137 (14)	0.813
NSAIDs	63 (24)	283 (28)	0.204
Analgesic medications (n, %)			
Acetaminophen	34 (13)	51 (5)	<0.001
Opioid type pain relievers	40 (15)	76 (8)	<0.001
Over the counter supplements for gout (use in the past week) (n, %)			
Cherry Juice	21 (8)	111 (11)	0.161
Vitamin C	59 (23)	225 (22)	0.927
Turmeric	4 (2)	24 (2)	0.405
Chronic gout medications in those who are candidates			
Candidates	164	558	
Any ULT use	127 (77)	488 (83.0)	0.103
Allopurinol	84 (51)	378 (64)	0.002
Febuxostat	42 (26)	95 (17)	0.002

^a^ *NSAIDs* nonsteroidal anti-inflammatory drugs, *ULT* urate lowering therapy

## Discussion

Using a national gout registry, we were able to compare women and men with gout and identify differences in patient profiles with respect to gout-related burden of comorbid disease, contributing medical conditions, medications and diet. Even after using PS trimming techniques to balance the patient populations in terms of age and duration of gout, women had a greater burden of musculoskeletal and medical comorbid illness as well as functional impairment. Factors that contribute to gout, including obesity, diuretic medications and diet differed by sex. Women more often had renal disease, and concomitant use of thiazides and/or other diuretics. In contrast, men were more likely to report intake of foods associated with both the development of gout and gout flares, including beef, pork, seafood, and alcohol. The most striking difference was seen in the intake of alcohol. This does reflect national trends in the US that demonstrate that men consume greater quantities of alcohol overall as compared to women with beer as the top choice for men while women drinkers have a preference for wine [14].

Our study is similar to others demonstrating women with gout to be older with a greater burden of comorbid illness, and less likely to have their disease proven by crystal diagnosis [6–8]. It is unclear whether lack of crystal confirmation was due to women being less likely offered the procedure versus more likely to decline having it performed. Alternatively, the presentation of women might have been more definitively consistent with the diagnosis of gout and thus the rheumatologist did not pursue further testing. There were differences in the use of anti-inflammatory and analgesic medications, with women were more likely to be receiving acetaminophen and narcotics. However, these medications may have been used for other arthritic or painful conditions.

While patients of both sexes should receive care consistent with “best practices”, our observations can help providers to prioritize which factors to explore in terms of contributing to gout in men and women. We anticipate that that patient sex will influence the management of gout. For women, the greater prevalence of increased BMI and diuretic use in this subset suggests this will need to be targeted for intervention. Weight loss has been shown to decrease gout flares and can improve the management of the associated comorbid conditions such as osteoarthritis and diabetes [15, 16]. For men with gout, a modification of urate-generating dietary intake will need to be pursued given the association with gout flares consistent with treatment recommendations [13, 17–19].

An important strength of this study is that the registry includes rich clinical data on a national sample of gout patients cared for by rheumatologists. Limitations include the study cohort may not be a representative sample of US gout patients, although that seems less likely given the comparisons to other national gout populations [8]. Since this is not an inception cohort, we also could not assess the true baseline characteristics of patients at the time of gout diagnosis. However to address this limitation, we used PS methodology to explore the impact of imbalance in age and disease duration between the sexes and found similar findings.

## Conclusions

In conclusion, this national cohort demonstrates that the gout patient profile differs in women as compared to men. Potentially modifiable factors such as BMI, diuretic use and diet differ by sex. Additionally, the coexistence of comorbidities that influence the management of the condition, such as renal disease and diabetes, are much more common in women as compared to men. A better understanding of sex differences in gout patient profiles will provide a foundation for tailored treatment recommendations by providers.

## Additional files

Additional file 1: Table S1. Baseline characteristics. (DOCX 15 kb)
Additional file 2: Table S2. Dosing of urate-lowering therapies among eligible patients. (DOCX 12 kb)
Additional file 3: Figure S1. Comparison of the dietary servings among women and men with gout. The *p* values represent adjusted analyses adjusting for age, BMI, duration of gout, comorbidity burden [hypertension, diabetes, renal disease, hyperlipidemia], HCTZ use, other diuretic use and current use of a urate-lowering drug. (TIF 320 kb)

## Abbreviations

BMI: Body mass index
CKD: Chronic kidney disease
CORRONA: Consortium of Rheumatology Researchers of North America
HAQ: Health Assessment Questionnaire
NSAIDs: Nonsteroid anti-inflammatory drugs
PS: Propensity score
SUA: Serum urate levels
ULT: Urate-lowering therapy
