# Supplementary material for: Sex differences in gout characteristics: tailoring care for women and men
Source: BMC Musculoskelet Disord. 2017 Mar 14;18:108. doi: 10.1186/s12891-017-1465-9 (PMC5351188; doi:10.1186/s12891-017-1465-9)
Supplement: Additional file 1: Table S1. — Baseline characteristics. (DOCX 15 kb) [file 12891_2017_1465_MOESM1_ESM.docx]

**Table S1. Baseline Characteristics**

|  | **Original** | | | **Scenario 1** | | | **Scenario 2** | | | **Scenario 3** | | |
| --- | --- | --- | --- | --- | --- | --- | --- | --- | --- | --- | --- | --- |
| **Characteristics** | **Women** | **Men** | **P value** | **Women** | **Men** | **P value** | **Women** | **Men** | **P value** | **Women** | **Men** | **P value** |
|  | **N= 262** | **N=1011** |  | **N=228** | **N=917** |  | **N=228** | **N=861** |  | **N=185** | **N=484** |  |
| Age (mean years, SD) | 71.2 ±10.7) | 60.9 (±13.5) | <0.0001 | 69.5 (±8.9) | 60.7 (±12.5) | <0.0001 | 69.5 (±9.0) | 61.9 (±11.3) | <0.0001 | 69.5 (±6.9) | 66.3 (±7.3) | <0.0001 |
| Duration of gout (mean, SD) | 6.1 (± 7.1) | 11.0 (±9.8) | <0.0001 | 6.1 (±6.4) | 10.1 (±10.1) | <0.0001 | 6.1 (±6.4) | 10.1 (±8.4) | <0.0001 | 5.2 (±4.5) | 7.4 (±5.3) | <0.0001 |
|  | N=261 | N=1008 |  |  |  |  |  |  |  |  |  |  |
| Gout associated comorbidities (n, %) | | | |  |  |  |  |  |  |  |  |  |
| Heart Disease | 41 (16) | 147 (14) | 0.652 | 32 (14) | 128 (14) | 0.976 | 32 (14) | 124 (14) | 0.888 | 27 (15) | 87 (18) | 0.298 |
| Hypertension | 201 (77) | 574 (57) | <0.001 | 176 (77) | 519 (57) | <0.0001 | 176 (77) | 501 (58) | <0.0001 | 141 (76) | 308 (63) | 0.002 |
| Diabetes Mellitus | 73 (28) | 168 (17) | <0.001 | 65 (28) | 152 (17) | <0.0001 | 65 (28) | 149 (17) | <0.0001 | 57 (31) | 98 (20) | 0.004 |
| Renal Disease | 64 (24) | 136 (13) | <0.001 | 56 (24) | 117 (13) | <0.0001 | 56 (25) | 115 (14) | <0.0001 | 46 (25) | 76 (16) | 0.006 |
| Hyperlipidemia | 120 (46) | 394 (39) | 0.045 | 101 (44) | 356 (38) | 0.131 | 102 (45) | 347 (40) | 0.226 | 87 (47) | 222 (46) | 0.788 |
| Peripheral Arterial Disease | 2 (1) | 13 (1) | 0.485 | 2 (1) | 12 (1) | 0.596 | 2 (1) | 12 (1) |  | 2 (1) | 9 (2) | 0.479 |
