# Supplementary material for: Sex differences in gout characteristics: tailoring care for women and men
Source: BMC Musculoskelet Disord. 2017 Mar 14;18:108. doi: 10.1186/s12891-017-1465-9 (PMC5351188; doi:10.1186/s12891-017-1465-9)
Supplement: Additional file 2: Table S2. — Dosing of urate-lowering therapies among eligible patients. (DOCX 12 kb) [file 12891_2017_1465_MOESM2_ESM.docx]

**Table S2. Dosing of urate-lowering therapies among eligible patients.**

| Drug | Women | Men | P value |
| --- | --- | --- | --- |
| Allopurinol (mg), (n, %)  50-150  200-250  300-350  400-450  500+ | N=149  42 (28)  26 (17)  72 (48)  5 (3)  4 (3) | N=722  103 (14)  85 (12)  413 (57)  81 (11)  40 (6) | <0.001 |
| Febuxostat (mg), (n, %)  20-40  60-80  Other | N=53  34 (64)  18 (34)  1 (2) | N=138  66 (48)  60 (43)  12 (9) | 0.067 |
| Pegloticase, (n, %)  Yes  No | N=262  0 (0)  262 (100) | N=1011  8 (1)  1003 (99) | 0.149 |
| Probenecid (per day) (n, %)  250-500  750-1000  1250-2000 | N=5  4 (80)  1 (20)  0 (0) | N=16  10 (63)  3 (19)  3 (19) | 0.572 |
