# Supplementary figures and images for: Sex differences in gout characteristics: tailoring care for women and men
Source: BMC Musculoskelet Disord. 2017 Mar 14;18:108. doi: 10.1186/s12891-017-1465-9 (PMC5351188; doi:10.1186/s12891-017-1465-9)

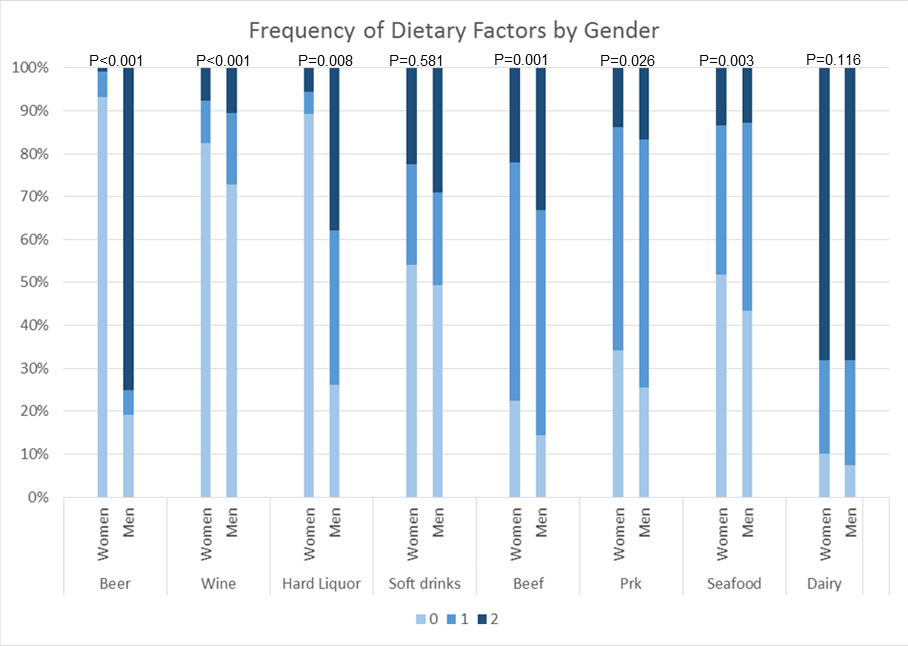

Supplement: Additional file 3: Figure S1. — Comparison of the dietary servings among women and men with gout. The p values represent adjusted analyses adjusting for age, BMI, duration of gout, comorbidity burden [hypertension, diabetes, renal disease, hyperlipidemia], HCTZ use, other diuretic use and current use of a urate-lowering drug. (TIF 320 kb) [file 12891_2017_1465_MOESM3_ESM.tif]
